# Scanning Electron Microscopy Study of Dental Gutta-Percha after Cutting

**Published:** 2006-07-01

**Authors:** Saeed Asgary, Masoud Parirokh, Mohammad Jafar Eghbal, Jamileh Ghoddusi

**Affiliations:** 1 *Department of Endodontics, Dental Research Center, Dental School, Shahid Beheshti University of Medical Sciences, Tehran, Iran*; 2 *Department of Endodontics, Dental School, Kerman University of Medical Sciences, Kerman, Iran*; 3 *Department of Endodontics, Dental School, Mashad University of Medical Sciences, Mashad, Iran*

**Keywords:** Cutting, Gutta-Percha, SEM

## Abstract

**INTRODUCTION:** The purpose of this study was to evaluate the morphologic surface of gutta­percha cones after cutting with different methods.

**MATERIALS AND METHODS:** The apical 3 millimeters of forty standardized, gutta-percha cones size 40 were cut off using scissors or a scalpel against a glass slab. The samples were then examined under scanning electron microscopy (SEM) for topographic deformity.

**RESULTS:** According to results, cutting with scissors produced significant topographic deformity in the standardized gutta-percha cone surface but cutting with sharp surgical instrument against a glass slab allowed the development of a smooth and unmodified gutta­percha cone surface.

**CONCLUSION:** Results of this study recommended that cutting the tip of a gutta­percha point with a sharp scalpel against a glass slab would produce more reasonable surface morphology than using scissor for the same procedure.

## INTRODUCTION

The objectives of modem endodontic therapy are to clean and shape the root canal system to create a fluid tight seal along the entire length of the cavity (1). In other words, the root canal filling materials must occupy the whole space of shaped root canals, promoting an adequate seal in coronal, lateral, and apical aspects of the root canal system. Filling of the root canal system eliminates the empty space, perpetuating the status of disinfection obtained after the canal preparation and reducing the potential risks of reinfection (2).

The fitting of the master cone in the apical portion of the root canal plays a very important role in the attainment of an adequate apical seal. In addition, fitting is important in preventing the extrusion of the filling material through the apical constriction (3).

There have been many studies on the gutta­ percha cones used in endodontic therapy. The sizing of gutta-percha cones is based on having a similar size and taper as the endodontic files. However, studies have shown that there are variations existing between different brands of cones and discrepancies in the diameters of instruments and gutta-percha points of the same size (4-6). Thus; the fitting of the master cone is frequently obtained after cutting segments off the standardized gutta-percha point. Cones are usually clipped with scissors, sharp surgical instrument, or razor blade (7-9). It has been claimed that an irregular cut of the gutta-percha cone may cause an improper fit of the master cone and thus prevents the attainment of an apical seal (10).

The aim of this study was to investigate the surface morphology of standardized gutta­ percha cones after cutting them with two different methods.

## MATERIALS AND METHODS

Forty gutta-percha standardized cones size 40 (Ariadent, Asiachimiteb, Tehran, Iran) were used in this experiment. Cones were divided in 2 groups of 20 samples each. In group 1, the cones were clipped at 2-3 mm from the tip by means of a sharp surgical scissors (Asculap, Germany). In group 2, cones were cut with a sharp scalpel blade No.15 (Asculap, Germany) against a glass slab (B.D, Iran). Gutta-percha cones were then investigated without any coating process using a conventional and low­ vacuum scanning electron microscope (SEM) (Hitachi S2250, N, Japan). Scanning electron micrographs of the gutta-percha cones were evaluated by three endodontists, according to the following criteria. Morphological event was evaluated by scores of 1 to 2, with 1 being the supenor.

Cutting surface:

1- Presence of one plane

2- Presence of two planes

Flange:

1- Absent

2- Presence of one or two flanges

Burs:

1- Absent

2- Present

## RESULTS

**Figure 1:**
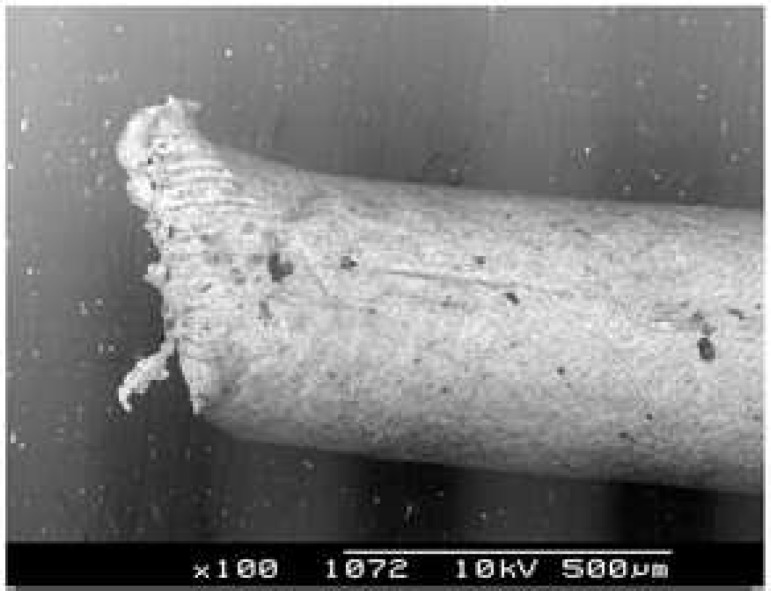
Scanning electron micrograph of Gutta-Percha Cone cutting with scissors. There are two plane and convergent flange (original magnification × 100).

**Figure 2:**
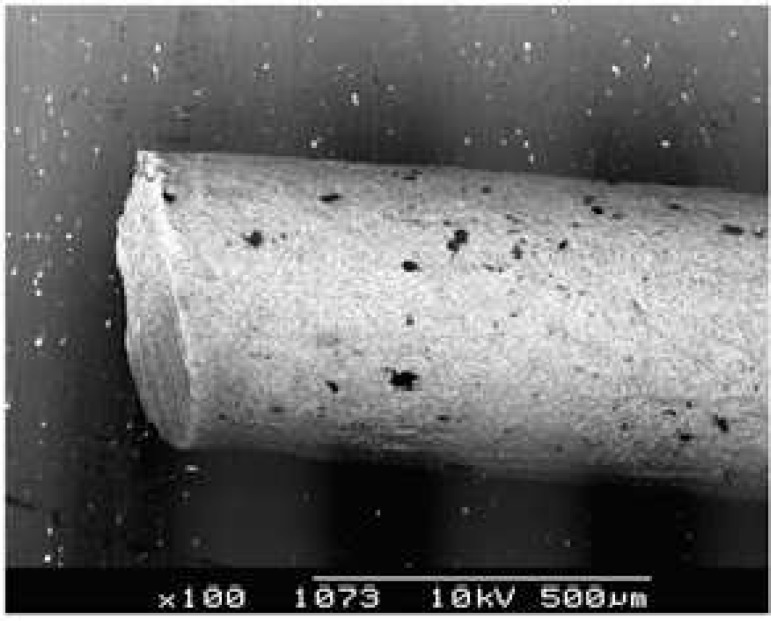
Scanning electron micrograph of Gutta-Percha Cone cutting with sharp surgical instrument against a glass slab. Regular cut surface (original magnification × 100).

Investigation of the standardized gutta-percha cones after two different cutting procedures revealed different features. In group 1, the cut surfaces of all samples showed two plane, one or two convergent flanges and burs (Figure 1). In group 2, gutta-percha cones showed smooth surfaces after cutting with sharp surgical instrument (Figure 2).

## DISCUSSION

Cutting of gutta-percha cones with sharp surgical instrument or scissors is commonly necessary during the master cone -fitting procedure. Discrepancies induced in the cone tip after cutting may theoretically impede a good adaptation in the apical portion of the root canal. In consequence, inadequate master cone fitting may compromise the attainment of the apical seal provided by the root canal filling. The cut of the gutta-percha cones occurs by the application of a shear stress in the transverse section of the cone. The cutting procedure induces an elastic compression of the material.

**Figure 3:**
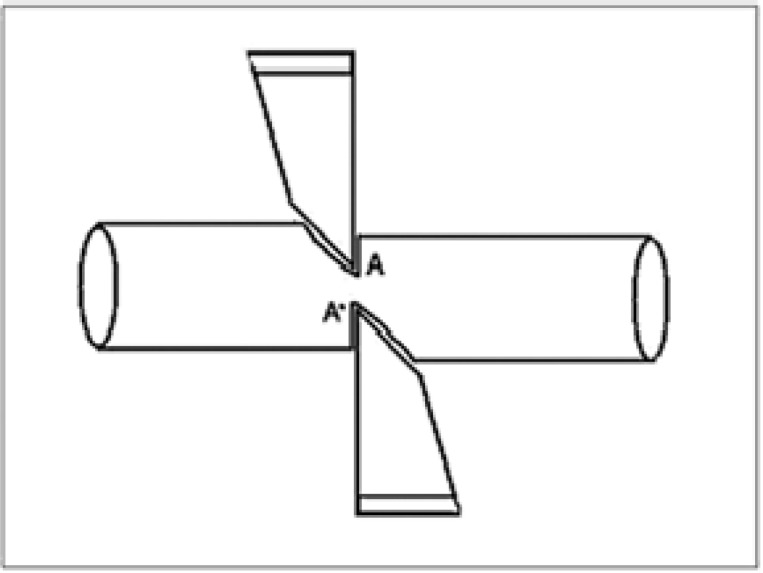
Schematic figure of the load applied to the cone during cutting with scissors.

Close to the cut surface, cone fibers bend in a similar direction to the movement applied to the cutting instrument. When the applied pressure overcomes the elastic resistance of the material, the cone is separated in two parts. The cone fibers bending during the cut show a plastic deformation in the whole cross-section of the material (8). The results of this study revealed significant differences in cone surfaces after two different cutting methods. In group 1 (scissors), the cut surfaces of all samples showed two plane, one or two convergent flanges and burs. The magnitude of cone deformation after cutting was dependent on both scissors shape and cone plasticity. Figure 3 shows the form of the loading applied on the cone during cutting with scissors. At points A and A', plastic deformation of the cones occurs. As the load increases, the cone is moved through the two blade surfaces of the scissors, producing two different inclined planes (Figure 3). Other researchers have reported similar findings (7,9). In group 2 (sharp surgical instrument), all samples showed smooth cut surfaces. The cut using sharp surgical instrument created shear stress in the cross­ section of the cone, but the plane surface of the glass slab reduced the plastic deformation of the cone. Therefore, the stress tensions induced by the blade and the glass slab allow the development of a plane cut surface, which is perpendicular to the direction of displacement of the cut instrument. This procedure is recommended by other investigators (8-10).

## CONCLUSION

The results of this research showed that the tips of standardized gutta-percha cones can be regularly cut off using sharp surgical instrument against a glass slab. Cuts using scissors produced irregular surfaces that might impede adequate master cone fitness.
